# High-Throughput Phenotyping (HTP) Data Reveal Dosage Effect at Growth Stages in *Arabidopsis thaliana* Irradiated by Gamma Rays

**DOI:** 10.3390/plants9050557

**Published:** 2020-04-27

**Authors:** Sungyul Chang, Unseok Lee, Min Jeong Hong, Yeong Deuk Jo, Jin-Baek Kim

**Affiliations:** 1 Radiation Breeding Research Team, Advanced Radiation Technology Institute (ARTI), Korea Atomic Energy Research Institute (KAERI), 29 Geumgu-gil, Jeongeup-si, Jeollabuk-do 56212, Korea; schang8@kaeri.re.kr (S.C.); hongmj@kaeri.re.kr (M.J.H.);; 2 Smart Farm Research Center, Korea Institute of Science and Technology (KIST), 679 Saimdang-ro, Gangneung, Gangwon-do 210-340, Korea; hello@drkumo.kr

**Keywords:** high-throughput phenotyping (HTP), mutation breeding, machine learning image, growth pattern analysis, dosage effect, gamma rays

## Abstract

The effects of radiation dosages on plant species are quantitatively presented as the lethal dose or the dose required for growth reduction in mutation breeding. However, lethal dose and growth reduction fail to provide dynamic growth behavior information such as growth rate after irradiation. Irradiated seeds of *Arabidopsis* were grown in an environmentally controlled high-throughput phenotyping (HTP) platform to capture growth images that were analyzed with machine learning algorithms. Analysis of digital phenotyping data revealed unique growth patterns following treatments below LD_50_ value at 641 Gy. Plants treated with 100-Gy gamma irradiation showed almost identical growth pattern compared with wild type; the hormesis effect was observed >21 days after sowing. In 200 Gy-treated plants, a uniform growth pattern but smaller rosette areas than the wild type were seen (*p* < 0.05). The shift between vegetative and reproductive stages was not retarded by irradiation at 200 and 300 Gy although growth inhibition was detected under the same irradiation dose. Results were validated using 200 and 300 Gy doses with HTP in a separate study. To our knowledge, this is the first study to apply a HTP platform to measure and analyze the dosage effect of radiation in plants. The method enabled an in-depth analysis of growth patterns, which could not be detected previously due to a lack of time-series data. This information will improve our knowledge about the effects of radiation in model plant species and crops.

## 1. Introduction

Food security issues are emerging globally due to climate change, population growth, economic growth in developing countries, and increasing demand for bioenergy [1]. In response, countries worldwide are developing genetic resources to strengthen intellectual property rights. Identifying beneficial a gene pools from plants with inter- and intra-species variation is key concept in plant breeding. Mutation breeding is a valuable alternative tool for addressing the diversity of problems associated with limited genetic resources [2]. Large mutant resources with various characteristics can be generated through mutagen treatment. Ionizing radiation is the most commonly used to generate useful mutations in plants because of the ease of treatment and high mutation frequency [3,4].

Selection of a desirable trait in a breeding population depends on utilizing phenotypic screening and genotypic markers. Phenotypic screening for useful mutants among numerous variations is the most important step for successful breeding. Traditionally, breeders visually phenotype plants when making their selections, which can be very subjective. To overcome these shortcomings, high-throughput phenotyping (HTP) systems were used to provide objectivity to the data. Breeding that involves the use of DNA and other molecular markers, or “marker assistant breeding,” has been widely practiced by plant scientists and breeders [5,6]. The rapid development of next generation sequencing (NGS) analysis tools has increased the use of NGS marker systems in large-scale commercial breeding schemes [7]. Unlike massive NGS-based genotyping technologies, plant selection for breeding began with high throughout phenotyping technologies [8].

Phenotypic data analysis is time-consuming and labor intensive. In some cases, the quality of the data is questionable and the whole volume of data points acquired through such practice is very limited [9]. This phenomenon is known as “phenotyping bottleneck” and the problem persists for selection in all biological systems [10]. To solve this problem, high-throughput phenotyping (HTP) and phenomic approaches were proposed [11]. The novel approaches incorporate multiple sensors on a mechanized platform to obtain plant growth data [12]. Large-scale studies performed using HTP have led to the discovery of new quantitative trait loci. For example, soil salinity inhibited plant growth and the stress response was quantified and compared between treated and non-treated of whole volume from convex hull area of rice [13].

Use of gamma rays accounts for half of the mutant plant varieties registered in the Joint FAO/IAEA mutant variety database [14]. Gamma irradiation penetrates tissues and affects mutation through formation of ions. These ions induce chemical reactions and damage chromosomes and DNA. This damage induces mutations in plant genomes. The biological effects of ionizing radiation depend mainly on the amount of energy absorbed by the biological system. 

The dosage effect of gamma radiation has been well documented in various plant species [15,16] including the model plant *Arabidopsis thaliana* [17]. The studies uncovered correlations between acute gamma ray dosage and survival rate, expressed as mean lethal dose (LD_50_), in many plant species. In addition, Yamaguchi et al. determined the relationship between total gamma ray doses and dose rates [18]. A proper dose of gamma radiation based on LD_50_ was common practice for mutation breeder and scientist [19]. Phenotypic data combined with NGS data have been used to determine DNA variations in the genome of *Arabidopsis* [20]. However, the phenotyping bottleneck persists in mutant breeding programs and cutting-edge phenotyping tools are needed to alleviate the problem [21].

Mutant phenotypes from ethylmethanesulfonate (EMS)-induced *Arabidopsis* populations have been identified and Berná et al. determined that the phenotypes are inherited [22]. The relationships between genes and morphological changes were identified in EMS-induced mutant populations [23]. *Arabidopsis* seeds or seedlings were exposed to gamma rays [24] and carbon ions to generate various mutants [25,26]. However, there is a lack of corresponding time-series phenotypic data and information about the growth pattern at M_1_ that might hold subtle information but important information for plant scientists and mutation breeders.

The main objective of this study was to analyze data extracted from digital images taken on a HTP platform to compare subtle morphological responses to gamma-irradiation at different doses in M_1_ seedlings of *Arabidopsis* and to determine the most significant time intervals and phenotypic characteristics for dosage effects.

## 2. Results

### 2.1. Test of HTP Data for Severely Affected Phenotype

The main purpose of the preliminary trial was to test our image analysis [27] pipeline (Figures S1 and S2) to translate image data into quantitative data, and then quantitative data, and then compare the quantitative data from plants with high doses of radiation (600 and 800 Gy) to those with lower doses (200 and 400 Gy), as adapted from Kim et al. [28]. LD_50_ value was determined in 641 Gy, and it was calculated by counting the number of surviving plants (Figure S3a). The 800 Gy dose was removed due to a low germination rate (<10%) and above the LD_50_ but the results from the 200, 400, and 600 Gy doses were analyzed (Figure S1). The average PA, defined as the growth rate following each treatment, was calculated and visualized with a Plotly library [29] in R [30]. The result indicated that PA provided clear quantitative results, which could be used to compare the dosage effects of gamma rays and the effectiveness of the HTP platform to measure 350 samples (Figure S3b) with high confidence (Figure S2).

### 2.2. Growth Pattern Following the Four Radiation Doses Used in the Main Study

**Figure 1:**
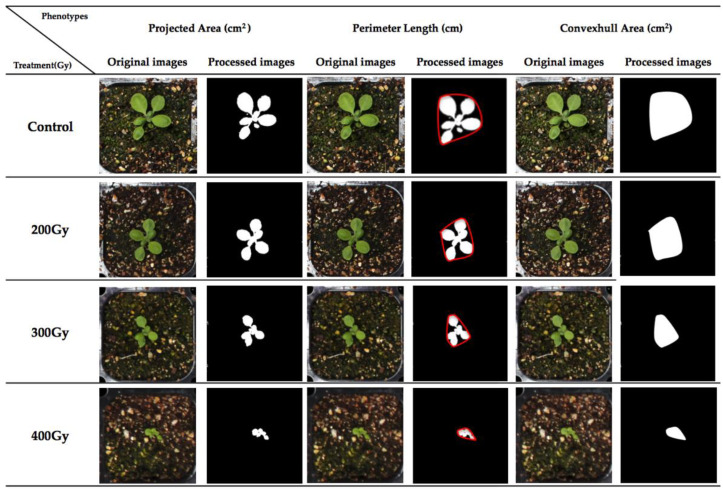
Definition of digital phenotypes and comparison among different gamma radiation doses at 17 days after sowing.

The purpose of the main study was to compare less severely affected groups of *Arabidopsis* plants irradiated with 100–400 Gy to detect small phenotypic differences in various time points (Figure S4) using the HTP platform (Figure 1).

**Figure 2:**
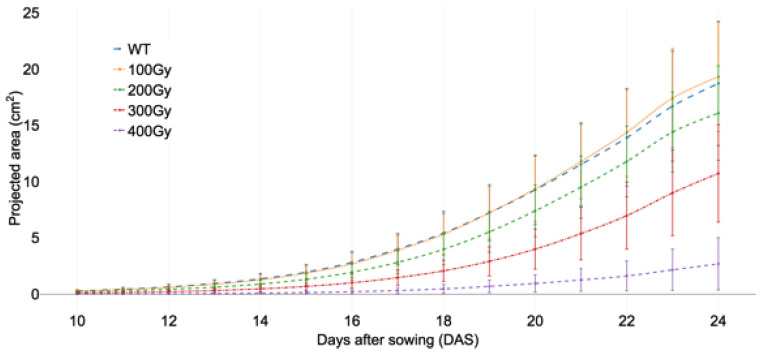
Comparing the growth pattern of *Arabidopsis* seeds irradiated at 100, 200, 300, and 400 Gy of gamma radiation (^60^Co). Results are means ± SD (*n* = 96). All treatments are shown as the average of 96 plants per treatment except 128 for wild type.

The PA dataset showed that two clustering groups emerged from the dataset and the group that underwent treatment over 300 Gy was separate from the control, 100, and 200 Gy groups (Figure 2). The growth pattern seen in the 100 Gy-irradiated group was indistinguishable from that of wild type (WT) *Arabidopsis* plants until 20 days after sowing (DAS) and beyond point was significant (*p* < 0.05). Daily growth changes were defined as absolute growth rate (AGR). The comparison of differences in leaflet sizes due to environmental interactions or treatments was defined as relative growth rate (RGR), involving the transformation of the whole leaflet area with natural logarithms between any given dates. The AGR of the 100 Gy group (2.11) was higher than that of the WT (2.06) (Table S2) and beyond 21 DAS the 100 Gy group displayed a better overall growth rate than the WT (Figure 1). The AGR of the 200 Gy group, however, never exceeded that of the WT and the separation in growth pattern between the WT and 200 Gy group widened until 19 DAS, after which the separation was constant (Figure 1). The AGR of the 300 Gy group was 2.07 at 23 DAS, which was similar to that of the WT at 20 DAS; thus, there was a gap of three days between the WT and 300 Gy groups in terms of AGR. At 23 DAS, the AGR of the 400 Gy group was 0.477 (Table S2) and this result was similar to that of the WT at the early vegetative stage (14 DAS). This result indicated that a dose of 400 Gy severely affected growth mechanisms (Figure 1).

### 2.3. Developmental Stages Selection for Statistical Analysis

**Figure 3:**
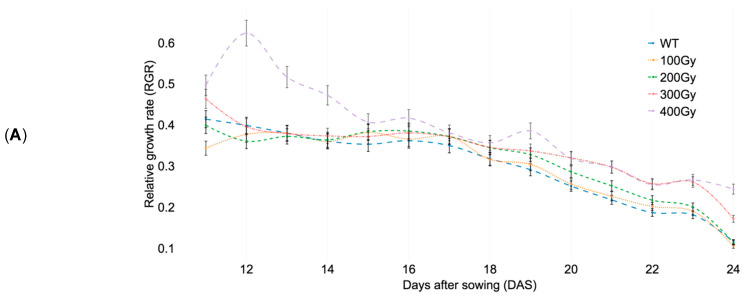

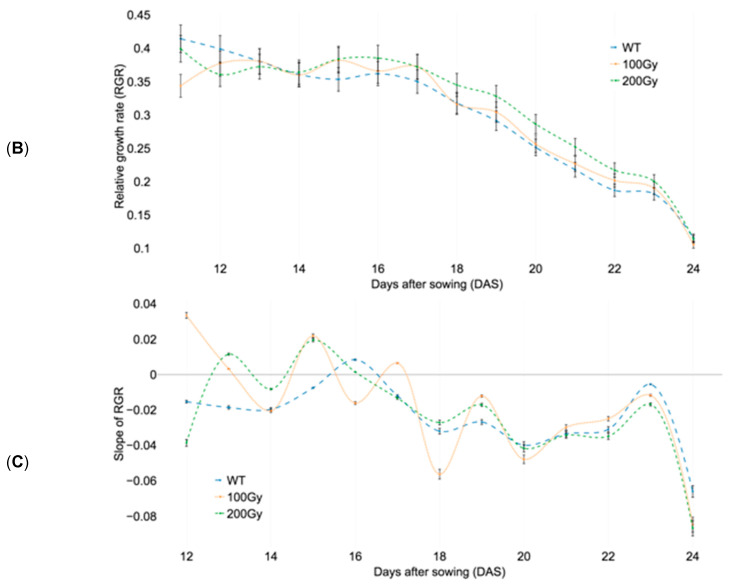
Comparing relative growth rate (RGR) of *Arabidopsis* seeds irradiated at 100, 200, 300, and 400 Gy of gamma radiation (^60^Co). Results are means ± SD (*n* = 96). (**A**) All treatments are shown as the average of 96 plants per treatment except 128 plants for wild type. (**B**) Only 100 and 200 Gy treatments are shown. (**C**) Slope of RGR.

There were differences in PA among the treatments and RGR was utilized to compare growth (Figure 3a) in relative terms [31]. The RGR values of the WT, 100 Gy, and 200 Gy groups were analyzed (Figure 3b). The RGR indicated that a uniform rate of growth was seen in the WT and 200 Gy group. The growth rate of the 100 Gy group fluctuated between that of the WT and the 200 Gy group between 14 and 19 DAS. In addition, the slopes of the RGR were visualized to check decreases or increases of the rate over all developmental stages. At least two treatments resulted in increased rates of growth at 14, 18, and 22 DAS, at which points two rosette leaves had emerged, 10 rosette leaves had emerged, and the first flower emerged, as defined by Boyes et al. [32]. Eighteen DAS represents the initiation of the reproductive phase [25] and the results showed that all of the rates increased; those increases seen in the WT, 100 Gy, and 200 Gy groups were similar to each other (Figure 3c). This result indicated that the growth mechanism associated with the reproduction phase was partially conserved, even when plants were treated with doses of 100 and 200 Gy.

### 2.4. Statistical Analysis and the Validation Study

Statistical analyses were performed at 14, 18, and 22 DAS to determine whether differences between the WT and the four groups exposed to different doses of radiation were different. First, PA data were used to separate the four dose groups at predetermined developmental stages. The differences between all four dose groups were found to be significant at *p* < 0.05 and a ranking conversion was observed between the WT and 100 Gy group at 14 and 18 DAS (Table 1).

In addition, the early growth stages (7–9 DAS) were selected to determine whether the leaflet area digital phenotyping data were separated by dosage effects. Statistical differences in PA were detected as early as 7 DAS among the four dose groups, except the 100 Gy group (Table 2). Second, CA, and PL data were utilized to separate treatments at the same developmental stages. The main reason was that CA and PL included geometric information (Table 1) and the graph showed similar but not identical trends for each parameter (Figure S5). As a result, CA and PL provided extra phenotypic information to separate the dosage effects. At 14 DAS, PA, CA, and PL data were utilized to separate the WT from the 400 Gy group. At 18 and 23 DAS, the WT was separated from all treated groups, except the 100 Gy group. This result indicated the HTP platform-derived data could detect dosage effects as early as 18 DAS (Table 1).

A validation study was performed to confirm the main study result. This experiment proceeded identically to that of the previous experiment but the 100 Gy dose was removed, because it was only separable in the late developmental stage (at 21 DAS and after), and the 400 Gy dose was removed, because it severely inhibited the growth mechanism (Figure 3a, Figure S4, Table S2). Comparison of growth pattern of between the main and the validation studies showed similar trends (Figure S6). At 19 DAS, PAs of WT and 200 Gy were not different between the main and the validation studies (Table S1). For that reason, 19 DAS was selected and the WT was observed to be statistically separated from the 200 and 300 Gy groups and showed same ranking response with statistical significance with the main study (Table 3). A summary of 350 plants for the preliminary, 480 plants for the main, and 320 plants for the validation studies data is available in Table S3. The results confirmed that the HTP platform-based screening method can feasibly be used to detect dosage effects in rosette structures and crops.

### 2.5. Morphological Characteristics of 200 Gy Sub-Populations

The visual inspection of selected plant images showed a distinct rosette shape (Figure S7a) between the angles of first true leaves [33]. In WT, this angle was 165°; in 200 Gy treated plants, the angle was 130° (Figure S7c). Manacorda et al. reported morphological (rosette) changes in *Arabidopsis* [34]. We report a unique shape from 200 Gy irradiated *Arabidopsis*. Detection of this shape in M_2_ would be interesting.

## 3. Discussion

The development of *Arabidopsis thaliana* is well characterized and the detail of its developmental stages has been established [32]. However, the manual identification of growth stages in individual plants is labor intensive and time-consuming. The extraction of growth data from a large number of plants is, therefore, not feasible. For that reason, radiation research communities were interested in using phenomic approaches to measure morphological changes according to the effects of radiation [21]. To our knowledge, no study has previously used a HTP platform to measure the dosage effects of a radiation source.

There are many advantages to utilizing a HTP platform in plant sciences. HTP platforms are able to collect multi-sensor data with limited effort from researchers [12]. A large number of samples have been tested in vivo and cutting-edge image processes enable the precise and detailed plant measurements to be collected [27]. Additionally, the environmentally controlled platform avoids phenotypic variations caused by genotype–environment interactions and allows the study of a range of plants from model *Arabidopsis* specimens to field crops [8]. *Arabidopsis* has been used in various stress tests in this controlled environment [10,35], with rosette measurements being the most common phenotypic evaluation carried out [36]. In our study, PA, CA, PL were extracted from plant images and from these data the detailed growth pattern found under each treatment was constructed during the main and validation studies (Figure S6). The data were converted to established developmental stages and agreed with those reported in a previously published study [32], this HTP also produced reproducible results (Table S1) that agreed with another reproducible HTP study in rice [13]. Due to high reproducibility in detection of subtle phenotypic change, we expect our HTP system can be applied to study agronomical traits that have been key challenges in plant breeding. Especially, it may be useful in mutation breeding research in which substitutions in a few genes often result in subtle changes in phenotypes as shown in rice [37]. Study and validation of agronomical traits in the mutant populations with HTP might be feasible [13] even though response phenotyping data from various environments is required.

Mutant lines show unique growth patterns compared with WT *Arabidopsis* plants [36,38,39]. Severely affected groups including those receiving doses of 300 and 400 Gy emerged from the image analysis of the main study and the 400 Gy group showed very delayed growth patterns compared with the WT. The quantitative data indicated that 400 Gy severely affects growth-related mechanisms (Table S2). *Haber et al.* [40] performed a gamma plantlet assay to measure true leaves emerged after exposure to gamma radiation and found that plants treated with doses of 400 Gy or higher failed to produce non-embryonic leaves. These results explain the possible growth limiting mechanism observed in our study. Bleuyard et al. reported that differences were not detected between WT and 200 Gy-treated *Arabidopsis* plants [41]. In another study, subtle differences were observed, even though the growth stages used and measurement practices were different [42]. Using the HTP platform and data analysis, we found that the effect of 200 Gy was not severe but statistically significant compared with the WT. The difference from the Bleuyard et al. [41] report might be due to the measurement of different growth stages. Bleuyard et al. [41] measured plants in early developmental stages and found no effect of radiation, whereas we found a distinct effect in the late developmental stages using time-series data. Using HTP and cutting-edge image process pipeline (Figure S1), it was possible to separate a large number of samples with not only visually distinct plant morphologies but also subtle differences in plant morphology (Figure S4). Furthermore, HTP and image analysis tools to reveal more phenotypic characteristics might help in the selection of proper doses for generating traits of interest. Survival rate in M_1_ generation according to increasing doses has been mostly used as the sole reference for determination of optimal dose for mutagenesis [19]. However, in mutation population construction, the condition of M_1_ plants should be also considered for production of M_2_ seeds. The HTP system in the present study can additionally provide precise data for growth rate, which can be a more detailed reference to analyze plant condition as well as mutation frequency in M_2_ generation. In this study, the appropriate dose of ionizing radiation was selected as 200 Gy for *Arabidopsis thaliana* ecotype Landsberg (*Ler*) based on distinct, but not severely affected phenotype.

Previous studies showed no differences between WT and plants irradiated with 100 Gy in terms of shoot area [43,44,45]. The main roots of *Arabidopsis* were significantly reduced in a 100-Gy treated group [46] and this might be expressed as differences in rosette area of the plant in later developmental stages. In summary, the indication in previous studies that a dose of 100 Gy resulted in no differences may be due to limited time-series phenotyping data in various developmental stages. In this study, we captured the growth of many 100-Gy treated *Arabidopsis* plants over time intervals from 10 to 24 DAS in a controlled environment. Initially, the 100 Gy group showed an irregular growth pattern but then, interestingly, the PA was greater than that in the WT during the late developmental stages (21 DAS and beyond). This result can be explained partially by radiation hormesis [47], even though a late developmental stage effect was not reported [48]. Measuring phenotype in time-series data over the *Arabidopsis* life span provides new information [49] about hormesis-related mechanisms. This method could apply to study other important traits when traits expressed in certain time in a life cycle.

Well-established quantitative data about developmental stages is fundamental to studies involving DNA level responses [25] and phenotypic responses [50] to gamma radiation [21]. Gamma irradiation affects the early developmental stage of barley [51] and the growth rate of offspring from wild carrots [52]. These studies suggest that growth patterns and of dosage effects can be compared if continuous phenotyping data for irradiated plants is available. In this study, the unique growth pattern of each irradiated group was constructed from HTP platform-based data and this revealed that fluctuating growth patterns at doses of 100 and 200 Gy were similar to the pattern in the WT. When seeds were exposed to multiple doses of gamma irradiation, the growth at 18 and 22 DAS was less affected by the radiation. These developmental growth stages transitioned into the early reproduction stage and the results suggested that the reproductive phase might offer resistance to certain ranges of radiation. Real et al. summarized the radiation dose rate-response effect on mortality and reproduction in various plant species [53]. However, the scope of the study was very limited due to lack of time-series phenotype data. This new information provides tools for the genetic study of reproduction mechanisms by radiation. Furthermore, the method was utilized to establish stress response in various developmental stages in *Arabidopsis*. 

Mutants generated using various radiation sources have long and distinct histories in plant breeding [54]. The variations arise from different radiation sources and doses. In this study, we showed that dosage effects studied using the HTP platform generated precise phenotyping data for small difference detection in various developmental stages of M_1_ *Arabidopsis*. Mutant phenotypes of M_1_ [55] were studied but most studies focused on M_2_ [18,56] because of the distinct phenotypes in M_2_ [19]. Merging the phenotype with NGS data could detect minor quantitative loci (QTL) of traits of interest in M_2_ with subtle differences detected in the generation.

The HTP platform and data analysis procedure might be applicable for characterizing the dosage effect on small crop species such as micro tomatoes even though our studies only tested *Arabidopsis* plants. Furthermore, the same data analysis procedure with different HTP system such as plant-to-sensor type [57] enables characterization of the dosage effect on crop. Yamaguchi et al. discovered that the leaf length (indicating time intervals between growth stages) of plants is highly correlated to the DNA contents and mutant frequency [18]. We showed that the selection of a significant time interval for radiation dose was effective for detecting differences in irradiated seeds. Hence, a focus on limited time intervals might increase the chance of finding mutant phenotypes in a specific developmental stage at M_2_ and generations beyond. 

Phyllotaxis [33] describing arrangements among plant organs is a new concept among plant breeders. Previous studies in *Arabidopsis* discovered that phyllotaxis is associated with multiple genes [58,59]. Virus-infected *Arabidopsis* showed statistically different rosette shapes with HTP [34]. Additionally, the leaf angles of crops are controlled by multiple genes [60]. In this study, distinct rosette shapes were identified in a portion of 200-Gy irradiated plants in a series of data analyses. Many challenges arose in applying the phenotypic data to mutation breeding because of the difficulty in extracting accurate data from complex and overlapping characteristics, and in tracking individual plant structures, e.g., leaflets. However, image analysis is rapidly developing, especially for deep learning analyses [61], and we show promising results (Figures S1 and S2). Geometric information might be used in the future for phenotypic markers using information associated with agronomical traits and multiple environmental factors. 

The selection of desirable plants from populations is key to plant breeding but quantitative measurement was lacking in many breeding schemes. Field breeders make selections based on their personal knowledge and incorporate them into the breeding program. We showed that selection of a proper dose of gamma radiation from the subtle response of *Arabidopsis* was feasible, and this method could reduce the burden of screening multiple doses on plant samples. The HTP platform will enable the development of sophisticated breeding strategies based on precisely quantified characteristics, as determined by time-series analysis. The prediction of sorghum biomass from a height was made feasible [62] and yield prediction using time-series data [63] might be possible. Furthermore, mutant selection from tolerant gamma irradiated populations or resistant individual plant samples may be feasible. Merged with emerging HTP data [12] these results will assist in the field of radiation biology, because radiation can be used to develop many useful mutants, and their phenotypes can be screened by the HTP platform using quantifiable data. Effort and collaboration in radiation biology communities needs to move forward to embrace a digitalized radiation breeding scheme.

## 4. Materials and Methods

### 4.1. Gamma Irradiation

Dry seeds of *Arabidopsis thaliana* ecotype Landsberg (*Ler*) were irradiated with ^60^Co gamma irradiator (150 TBq of capacity, Nordion, Ottawa, ON, Canada) for 8 h at the Korea Atomic Energy Research Institute [64]. First, the seeds (first batch) for preliminary image analysis of phenotypic variation among treatments were prepared using four doses of gamma rays (200, 400, 600, and 800 Gy). Second, the seeds (second batch) for the main test of small phenotypic variation detection among treatments were prepared using four smaller doses of gamma rays (100, 200, 300, and 400 Gy). The second batch of irradiated seeds was used for validation study. All irradiated seeds were stored at 4 °C until further analysis.

### 4.2. Plants and Early Growth Conditions

Irradiated *Arabidopsis* seeds were sown in standard 32- or 50-cell seedling tray inserts (Famwin, Jeongeup-si, Republic of Korea) containing a 6:1:1 ratio mixture of soilless mixture, peat, and perlite. Seedlings were grown in walk-in chamber environmentally controlled at 22 °C under a photoperiod of 16/8 h (day/night). Each sown tray was covered with a transparent humidity dome, which was removed 5 days after sowing (DAS). The plants were grown for 7 days in total before being transferred onto the HTP platform.

### 4.3. Image Capture from the HTP Platform and Growth Conditions

The HTP platform was designed to capture multiple plant images in a time-series manner [27]. The plant images were programmed to be obtained every hour between 9:00 AM and 6:00 PM during the photoperiod. A motorized irrigation dripper was connected to each tray and filled with water every 4 days, over a 4-week period. LEDs provided (Lumens, Seoul, Republic of Korea) 16 h of lighting at 230 umol/m^2^/s. There were three experimental test sets generated from the HTP platform. All experiment dosages and irradiation sources are summarized in Table S3.

### 4.4. Image Analysis

Image analysis consisted of detecting the edges of the raw images and separating plant images from the background. A basic procedure for image analysis was adopted from Lee et al. [27] and updated for use in the present study. A summary of image analysis pipeline is available in Figure S1.

#### 4.4.1. Detection of Red Circle Indicator and Cropping into Individual Images

Raw images captured by a camera can be distorted due to the camera’s field of view. In general, this distortion can degrade the accuracy of image-based measurements (e.g., area and length).

Four pairs of points are needed to correct the distortion; x and y coordinates of four points in the source image and coordinates of four points in the target image (coordinates based on the actual length of a tray). In image analysis of plants, the four red circle indicators (the four points of the source image) were used to correct image distortion and as reference markers of actual size and were used in cropping individual plant images in the lab. The cropping can be accurately performed, and analysis errors can be minimized using the correction of image distortion (Figure S2c,d). However, at the experimental site, the red circle indicators were difficult to detect due to the noise associated with the soil and water. Thus, we designed deep learning-based robust red circle detection. RetinaNet was used for red circle detection [65] and the ResNet-152 network was used as a backbone for the feature extractor network [66]. A total of 2400 rectangular annotations of the red circle were used to train the detection network during the training steps. During analysis, four red circles were detected using the trained detection network and the center of each circle was calculated (Figure S2a). The distortion errors in images were corrected using the four center points and reference four points (predefined coordinates) (Figure S2b). Thirty-two corrected plant images were divided into individual plant images (Figure S2d).

#### 4.4.2. Separation of the Plant Image from the Background

Segmentation of a plant from background was very challenging until in recent years due to complexity of plant structure. Merged with machine learning algorithms like random forest (RF) to separate plant images from background images showed promising results [27]. However, deep learning-based algorithms showed excellent performance [67] over machine learning-based results. For that reasons, the U-net that was a network designed to segment biomedical images was selected for image segmentation. The network demonstrates high performance in segmenting small and thin objects and also shows good performance with few training data [67]. *Arabidopsis* is a small plant with thin petioles. Thus, in our study, we used U-net based pixel-wise semantic segmentation to separate the plant from the backgrounds.

The color images and mask images are needed to train a semantic segmentation network; the mask image consists of a black background and a white foreground. A total of 120 training data (i.e., color images and mask images of individual plants) were generated manually using the VIA annotation tool (Oxford, UK) [68]. Then, the generated training data were properly scaled and padded for the network size (512 × 512 dimensions) as preprocessing because the U-net requires a dimension size that is a multiple of 32 as an input; the original size of cropped original and mask images are 413 × 409 dimensions. Finally, the U-net based semantic segmentation network was trained using the processed training data.

The trained network output segmentation results (i.e., mask images). Then, the fully connected conditional random fields were applied to the segmented results for post-processing [69]; the post-processing method is known to produce a good performance for noise reduction of deep learning-based semantic segmentation. Finally, phenotype parameters such as projected area (PA), convex hull area (CA), and perimeter length (PL) of a plant (Table S3) were calculated using the processed segmentation images [36]. Furthermore, PA was utilized in comparisons with natural variation and physiological response in *Arabidopsis* [31,70].

### 4.5. Statistical Analysis

The dosage effects on the gamma irradiated population were tested for statistical significance using Duncan’s multiple range test, based on a significance level of *p* < 0.05. Statistical tests were performed in R [30] with Duncan’s test [71].

### 4.6. Molpholgical Characteristics of Gamma Irradiated Populations

Dosage effects on gamma irradiated populations were characterized using all phenotypic measurements (PA, CA, and PL) and principal component analysis (PCA) in R [30]. First, PCA was used to assess the main study results for WT and 200 Gy groups. Second, the same process was applied for the validation study. Two PCA results were compared, and examples were selected from the main study. One sample cluster shows WT as green square brackets and 200 Gy as purple square brackets (Figure S7a). Corresponding images were extracted and lines drawn along the edges of leaves and between the first true leaves (Figure S7c). Angles between the first true leaves were measured using the angle measurement function in ImageJ [72].

### 4.7. Data Analysis Procedure for the Main Study

The main study included time-series data with two dependent variables: irradiation dose and phenotypic data (Table S3). The data were analyzed using multiple steps due to the time domain. First, data were analyzed for PA in multiple doses to check general responses among treatments. Data analysis only utilized phenotype defined as PA and PA-derived information such as AGR. Second, selected time intervals and PA-derived RGR information were used to find similar responses to the four radiation doses during different developmental stages in *Arabidopsis*. Third, a statistical test was used to detect differences among the four doses at predetermined developmental stages with PA, CA, and PL. The data provided high confidence results (Figure S6) to detect small differences among treatments using the HTP platform (Figure 2).

## Figures and Tables

**Table 1:** Comparison of multiple digital phenotyping measurements at four doses of gamma rays on multiple days after sowing (DAS). Values in the same column followed by a different letter are significantly different (*p* < 0.05). PA: projected area, CA: convex hull area, PL: perimeter length.

DAS	Treatment (Gy)	PA (cm^2^)	CA (cm^2^)	PL (cm)
14	Control	1.3493a	5.8470b	10.1397b
	100	1.2675b	6.2346ab	10.8419a
	200	0.8963c	5.8655b	10.1553b
	300	0.4726d	6.6999a	11.0889a
	400	0.0929e	3.3721c	8.1944c
18	Control	5.3874a	11.6910b	13.4476b
	100	5.3385b	12.4183a	14.1398a
	200	3.9662c	10.6458c	13.0620c
	300	2.0589d	8.92156d	12.5973d
	400	0.4437e	4.50490e	9.3378e
23	Control	16.6866b	28.8198a	20.2470a
	100	17.3875a	29.0465a	20.2630a
	200	14.3426c	26.0152b	19.3870b
	300	8.9970d	17.9264c	16.4429c
	400	2.0390e	7.0165d	10.8608d

**Table 2:** Comparing the projected areas of four irradiated *Arabidopsis* populations on consecutive days after sowing (DAS). Values in the same column followed by a different letter are significantly different (*p* < 0.05). PA: projected area.

DAS	Treatment (Gy)	PA (cm^2^)
7	Control	0.0843b
	100	0.1079a
	200	0.0636c
	300	0.0306d
	400	0.0089e
8	Control	0.1256b
	100	0.1455a
	200	0.0907c
	300	0.0368d
	400	0.0098e
9	Control	0.1907b
	100	0.2205a
	200	0.1371c
	300	0.0560d
	400	0.0094e

**Table 3:** Validation experiments to measure multiple phenotypes with gamma dosages of 200 and 300 Gy. Values in the same column followed by a different letter are significantly different (*p* < 0.05). PA: projected area, CA: convex hull area, PL: perimeter length.

DAS	Treatment (Gy)	PA (cm^2^)	CA (cm^2^)	PL (cm)
19	Control	6.6751a	14.2777a	14.9116a
	200	5.6151b	13.1889b	14.4431b
	300	2.4909c	7.71947c	11.4332c

## Supplementary Materials

The following are available online at https://www.mdpi.com/2223-7747/9/5/557/s1, Figure S1: Image analysis pipeline of *Arabidopsis thaliana* for error correction, separated into individual plant images, segmentation of plant images, and extract phenotyping data. Panel A: Flow chart of image analysis pipeline. Panel B: Example images from each process. Figure S2. Correction of image distortion and error estimation of leaf area before and after correction. Panel a: raw image of tray. Panel b: images after image correction process. Panel c: result from image cropping process and error estimation of leaf area without correction. Panel d: result from image cropping process and error estimation of leaf area with correction. Figure S3: Comparing growth patterns of *Arabidopsis* seeds irradiated at 200, 400, and 600 Gy of gamma radiation (^60^CO). Results are means ± SD (*n* = 100). Figure S4: Comparing projected area (PA) of four irradiated *Arabidopsis* populations on multiple days after sowing (DAS). Figure S5: Comparing phenotypes of Arabidopsis seeds irradiated at 100 and 200 Gy of gamma radiation (^60^Co). Panel A: Projected area (PA). Panel B: Convex hull area (CA). Panel C: Perimeter length (PL). Figure S6. Comparing growth pattern of *Arabidopsis* irradiated seeds among the preliminary, the main, and the validation studies. Results were means of preliminary (*n* = 100), main (*n* = 120), and validation (*n* = 120) studies. The 200 Gy at preliminary study: Prel_200 Gy, wild type at main study: Main_WT, 200 Gy at main study: Main_200 Gy, wild type at validation study: Vali_WT, 200 Gy at validation study: Vali_200 Gy., Figure S7. Analysis of phenotypes (PA, CA, PL) with principal component analysis (PCA) of non-irradiated and irradiated (200 Gy) populations and selected phenotypes. Panel A: PCA result from the main study. Selected image of wild type plant is colored as green square box and 200 Gy plant as purple square box. Panel B: PCA result from the validation study. Panel C: Comparison of geometric shapes (red lines) of WT (left) and 200 Gy (right). Blue dashed lines indicate relative angles between the first two true leaves. Table S1: Comparison of multiple digital phenotyping measurements in three trials (preliminary, main, and validation) at 15, 19, 20 days after sowing (DAS). Values in the same column followed by a different letter are significantly different (*p* < 0.05). PA: Projected area. Table S2: Comparing absolute growth rate (AGR) of four irradiated *Arabidopsis* populations on multiple day after sowing (DAS). Results are means ± SD (*n* = 128). Table S3. Summary of all phenotyping data from preliminary, main, and validation studies.
