# Supplementary material for: High-Throughput Phenotyping (HTP) Data Reveal Dosage Effect at Growth Stages in Arabidopsis thaliana Irradiated by Gamma Rays
Source: Plants (Basel). 2020 Apr 27;9(5):557. doi: 10.3390/plants9050557 (PMC7284948; doi:10.3390/plants9050557)
Supplement: Supplementary file 1 [file plants-09-00557-s001.zip › Supplementary Materials_proof.docx]

**Supplementary Materials**


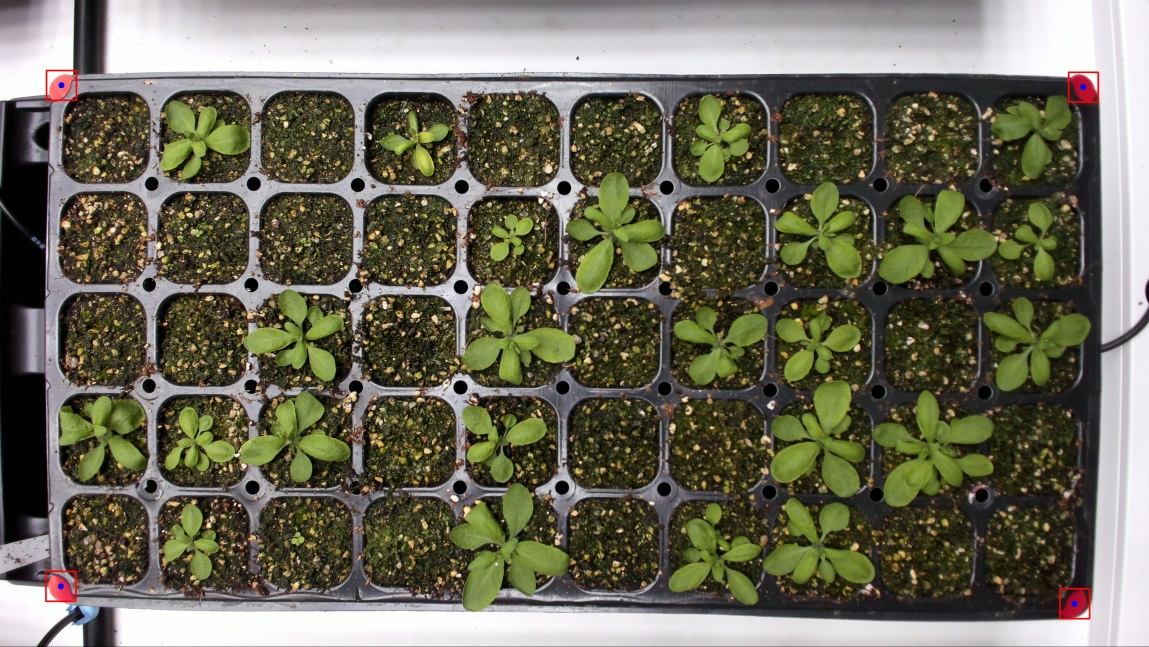


**
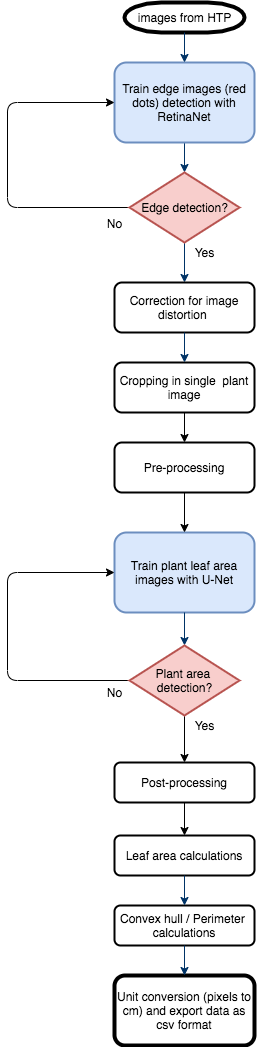

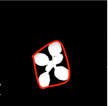

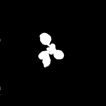

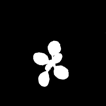

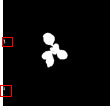

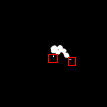

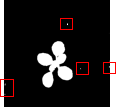

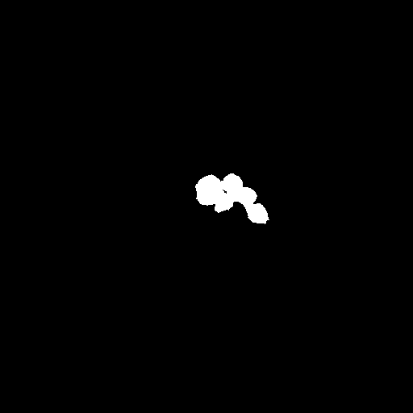

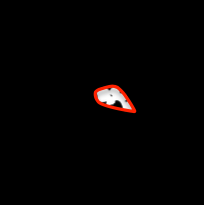

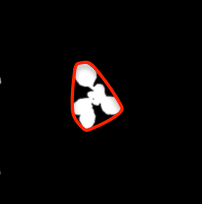

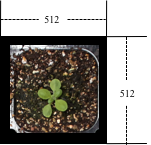

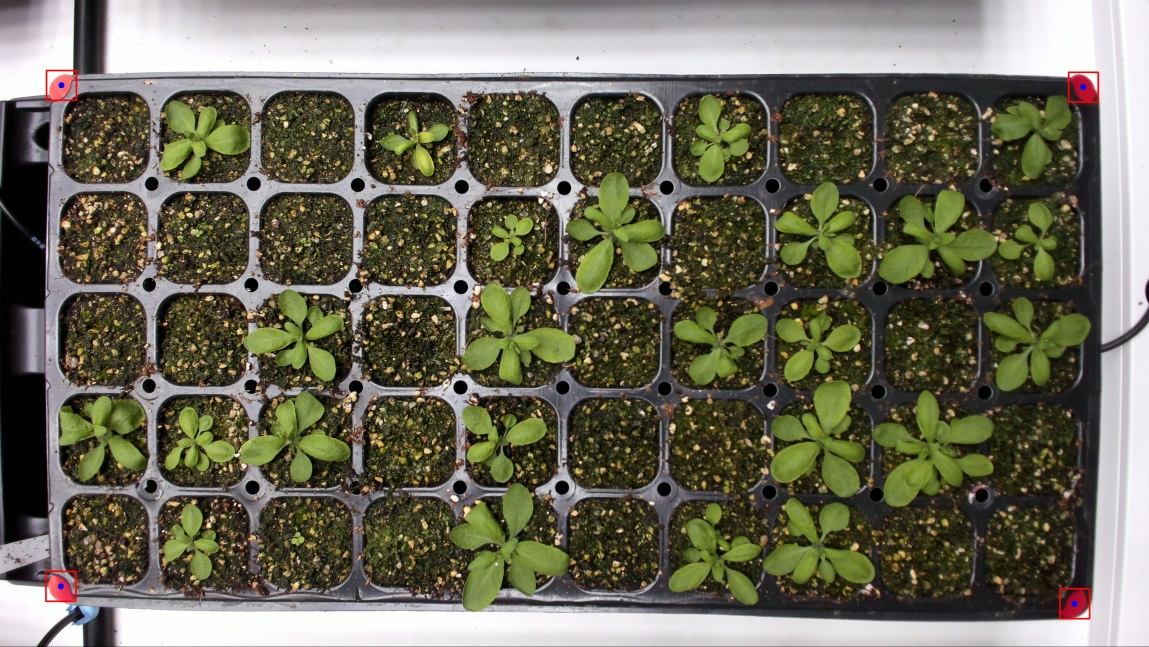

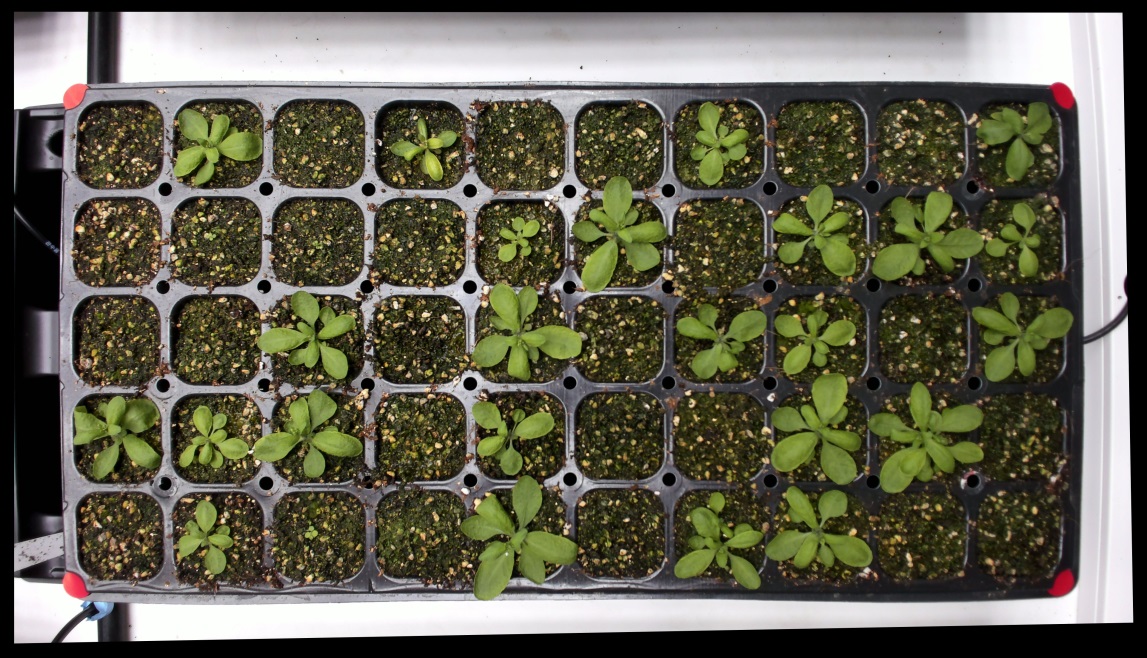

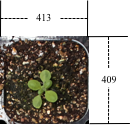
**
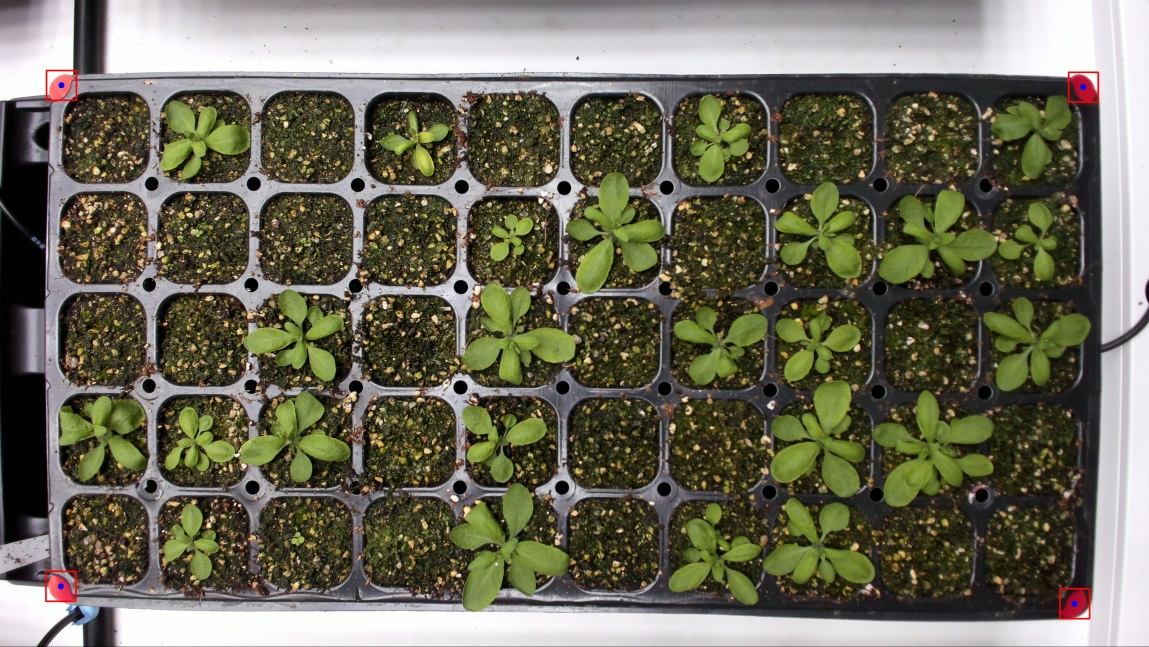
**
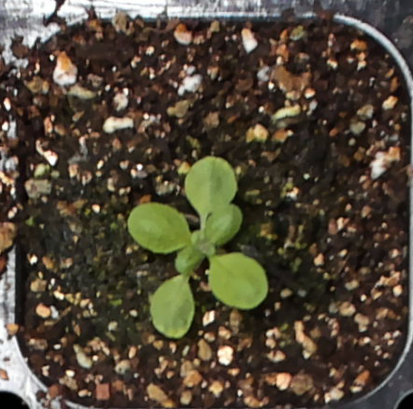

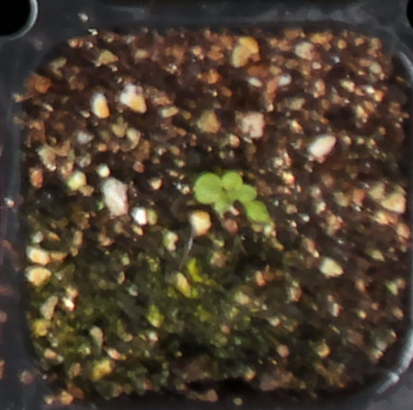

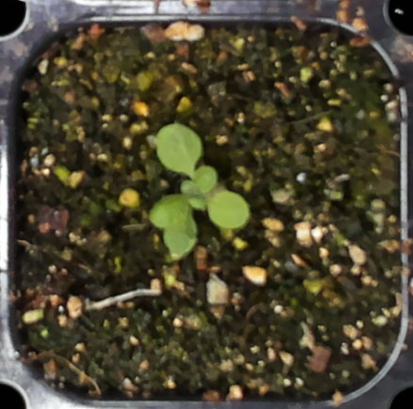
A B**

Fig. S1. Image analysis pipeline of *Arabidopsis thaliana* for error corrections, separate into individual plant images, segmentation of plant images, and extract phenotyping data. Panel A : Flow chart of image analysis pipeline. Panel B: Example images from each process.


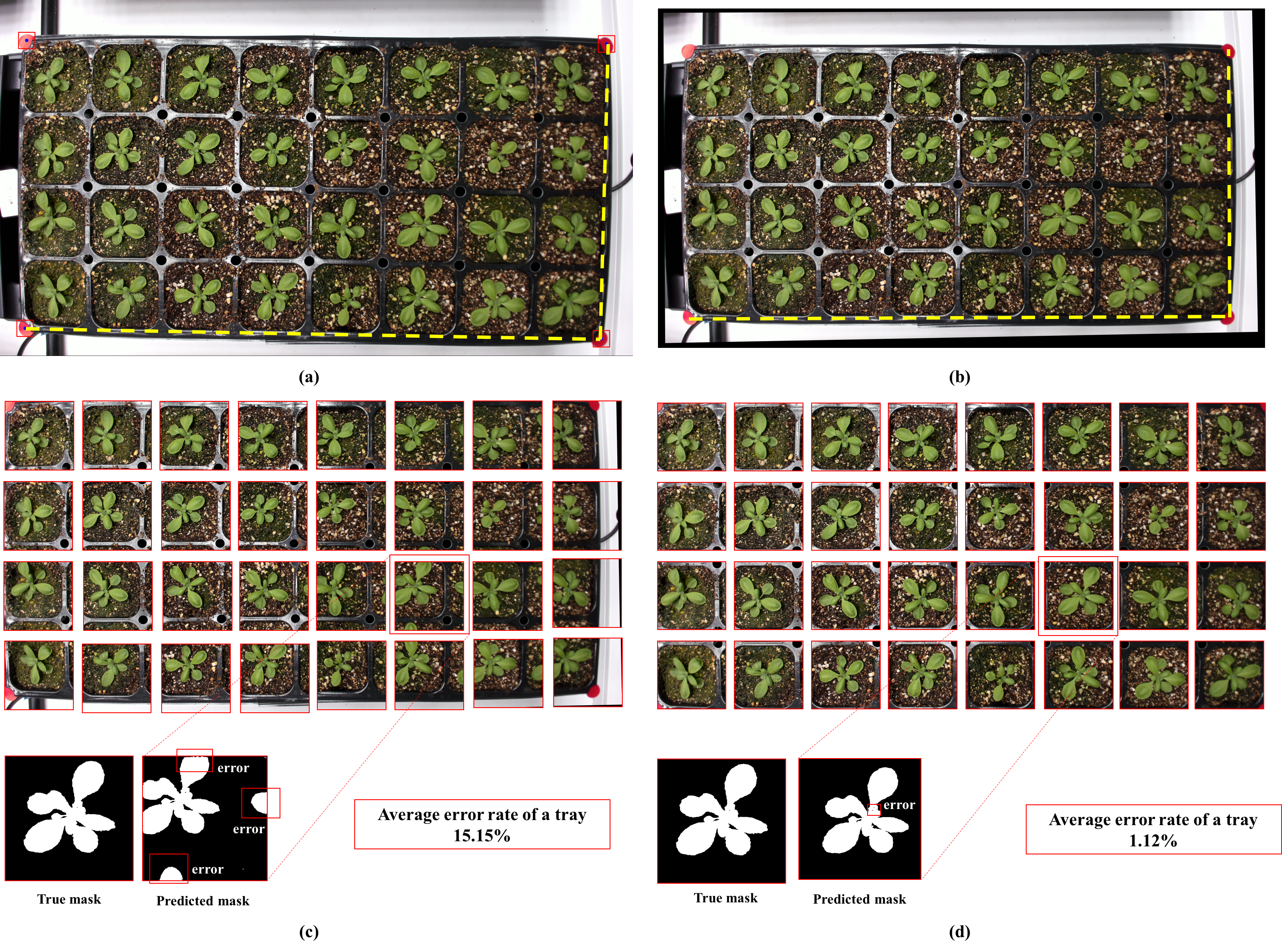


Fig. S2. Correction of image distortion and error of leaf area before and after correction. Panel a: a raw image of tray. Panel b: images after image correction process. Panel c: result from image cropping process and error estimation of leaf area without correction. Panel d: result from image cropping process and error estimation of leaf area with correction.

Fig. S3. Comparing survival rate (%) and growth pattern of *Arabidopsis* seeds irradiated at 200, 400, 600 and 800 Gy of gamma radiation (^60^CO). Panel A: Number of survival plants on gamma-rays and LD_50_ value. Panel B : Growth pattern comparison among 200,400, and 600 Gy irradiated plants. Results are means of 200 Gy (n=98), 400 Gy (n=90), and 600 Gy (n=32) ± SD.


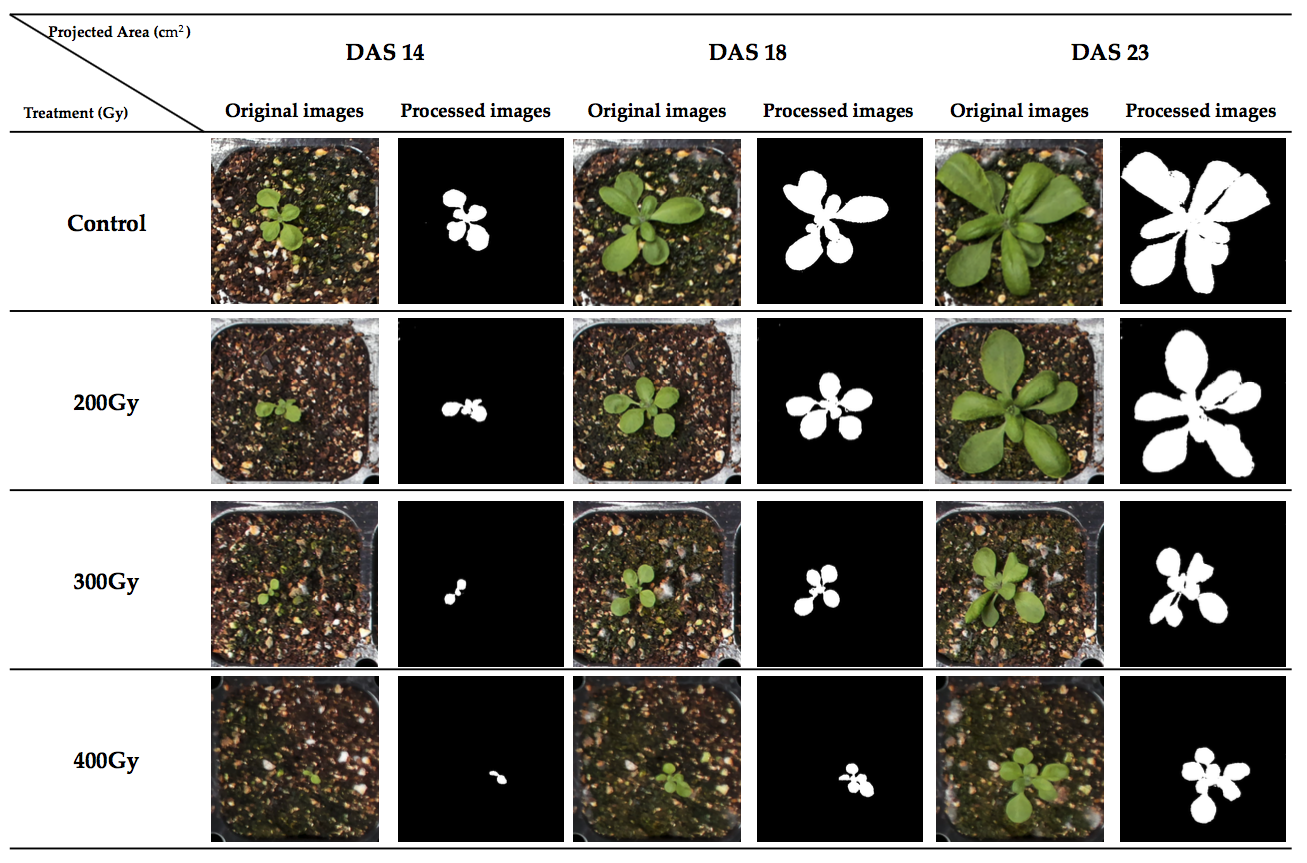


Fig. S4. Comparing projected area (PA) of four irradiated *Arabidopsis* populations on multiple days after sowing (DAS).

**
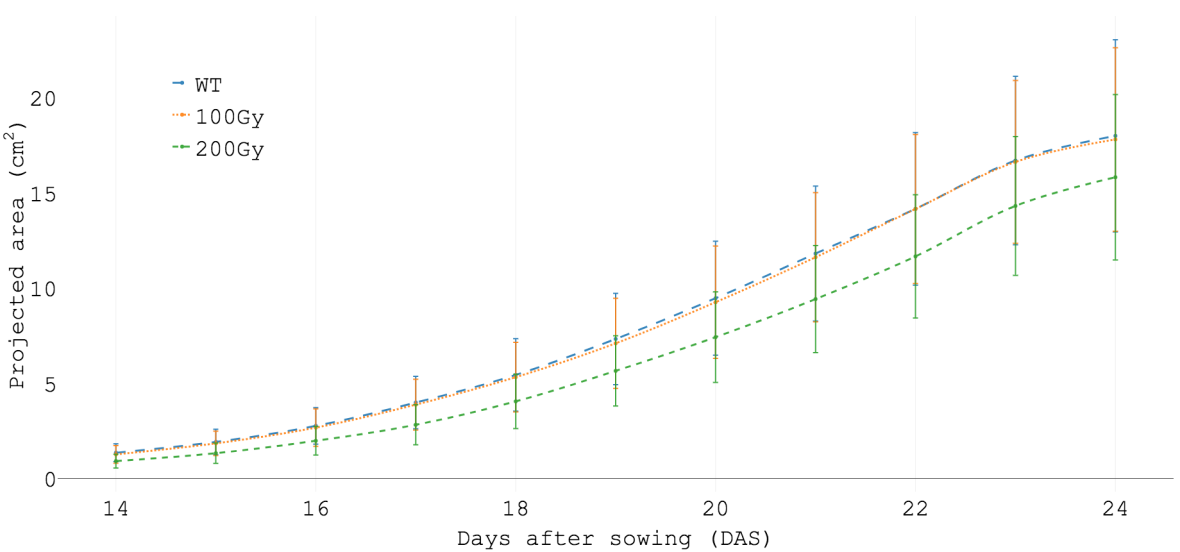
 A**

**
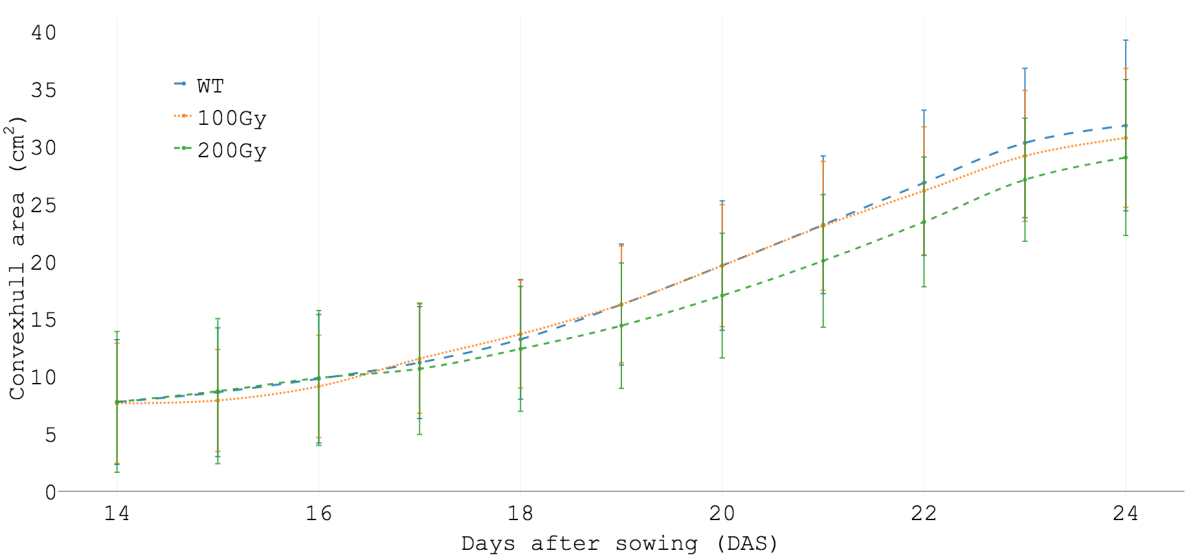
** **B**

**
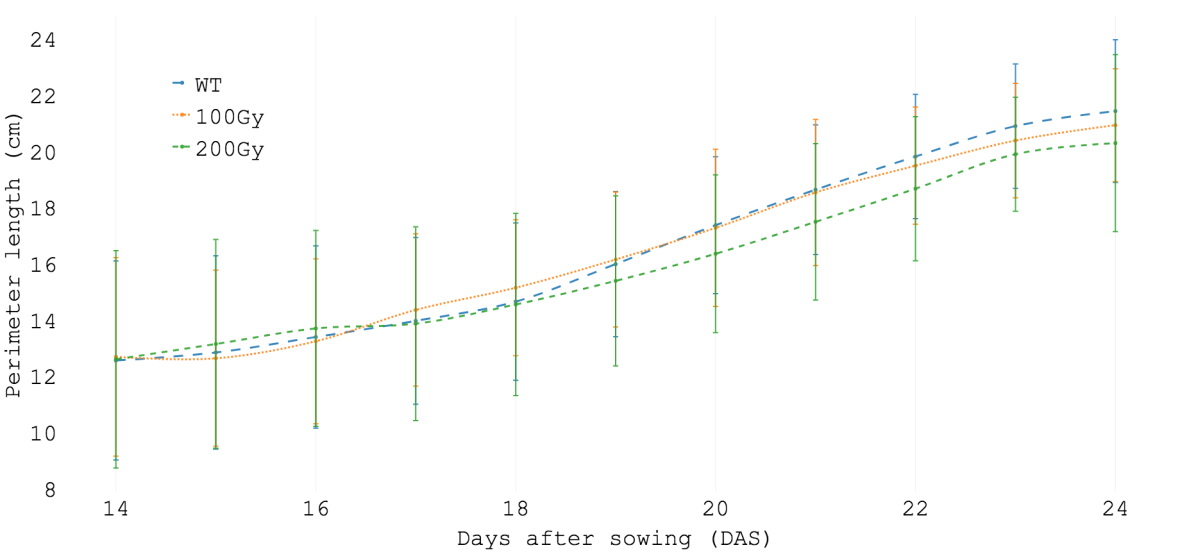
** **C**

Fig. S5. Comparing phenotypes of *Arabidopsis* seeds irradiated at 100 and 200 Gy of gamma radiation (^60^Co). Results are means ± SD (n = 64). Panel A: Projected area (PA). Panel B: Convex hull area (CA). Panel C: Perimeter length (PL).


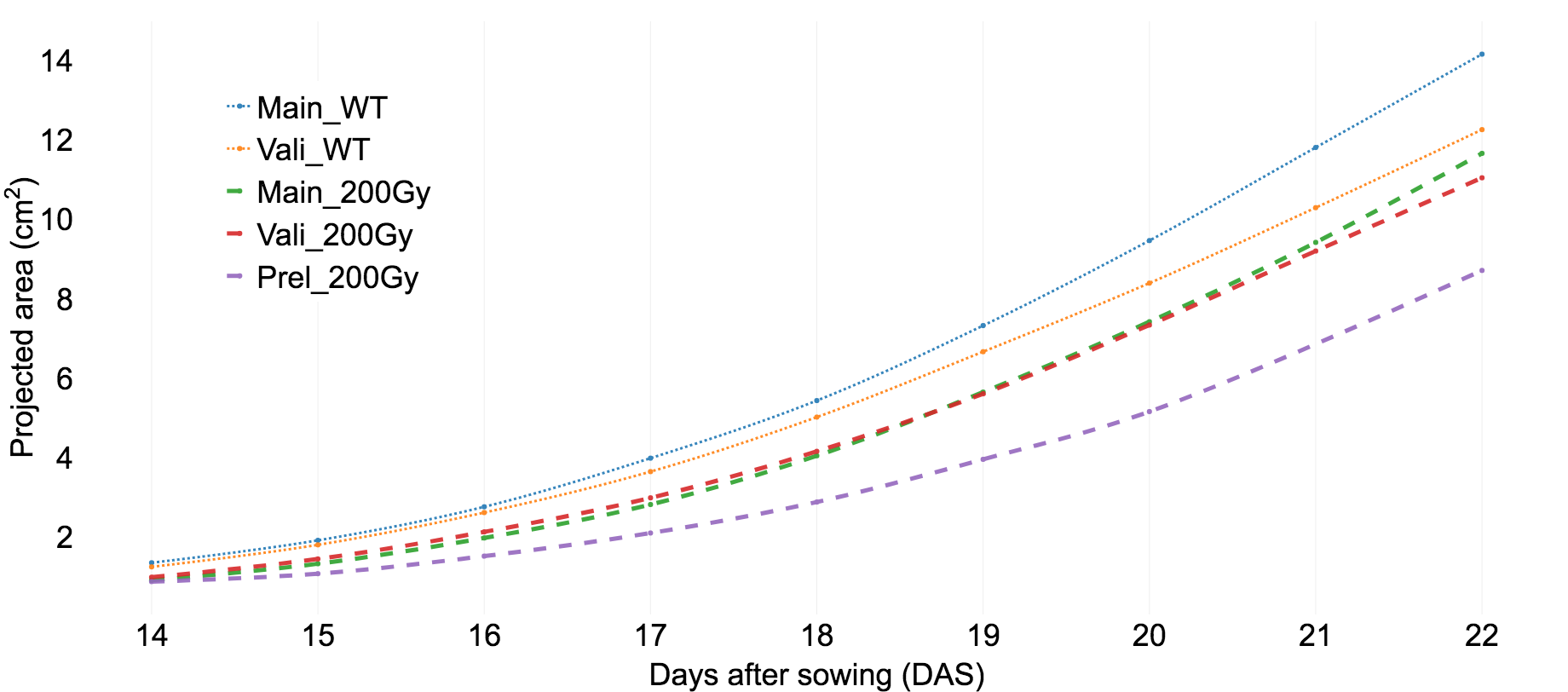


Fig. S6. Comparing growth pattern of *Arabidopsis* irradiated seeds among the preliminary, the main and, the validation studies. Results were means of preliminary (n=100), main (n=120), and validation (n=120) studies. 200 Gy at preliminary study: Prel_200 Gy, wild type at main study: Main_WT, 200 Gy at main study: Main_200 Gy, wild type at validation study: Vali_WT, 200 Gy at validation study: Vali_200 Gy.


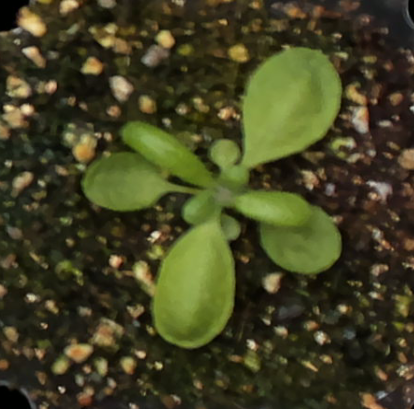
**
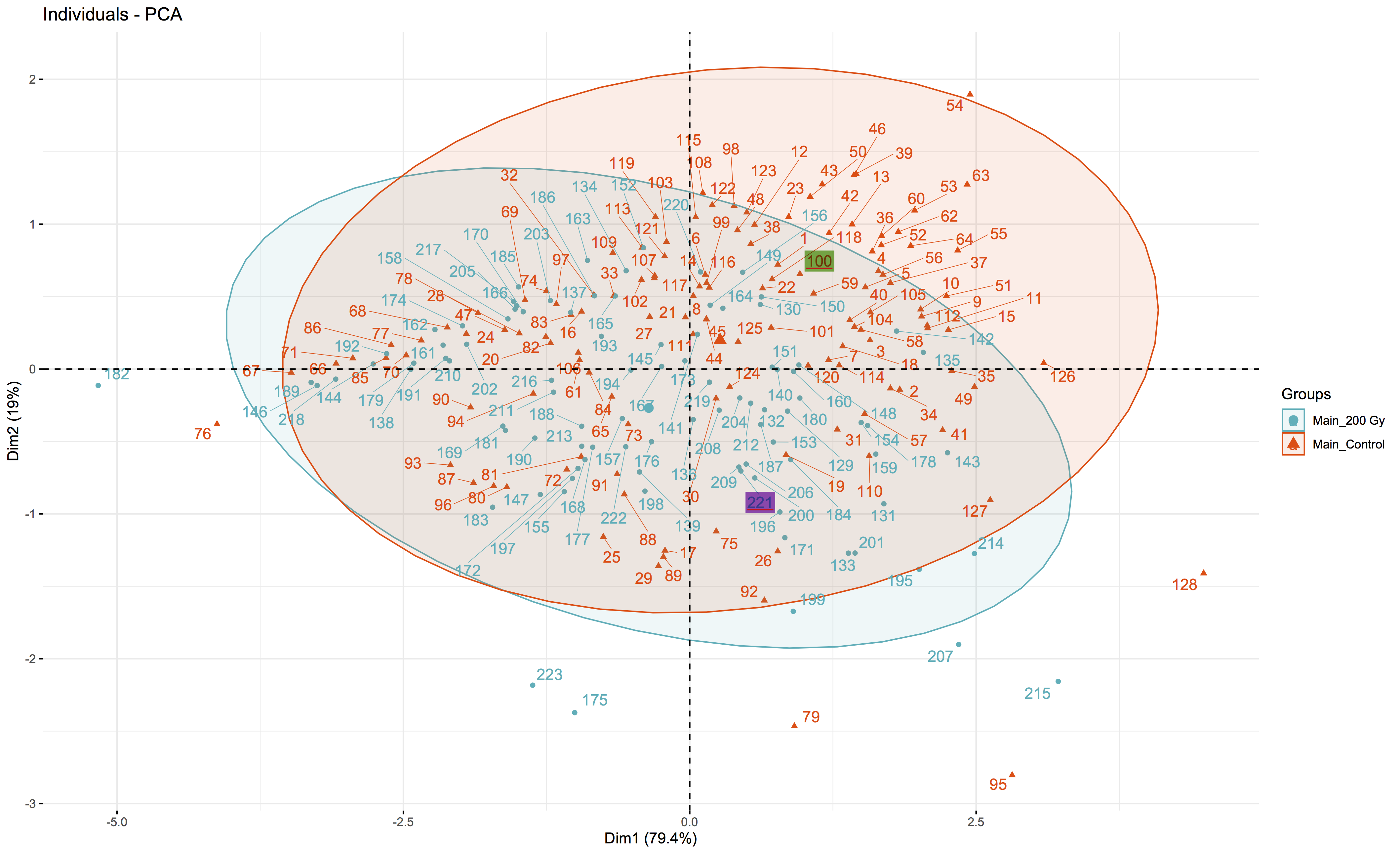
 A**

**
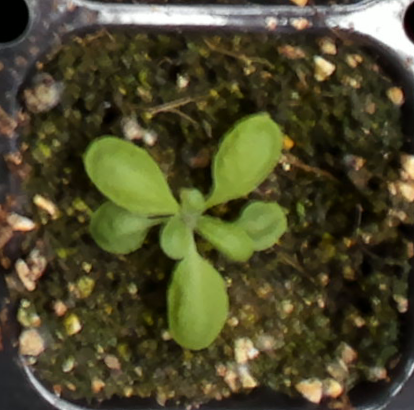
**

**B**


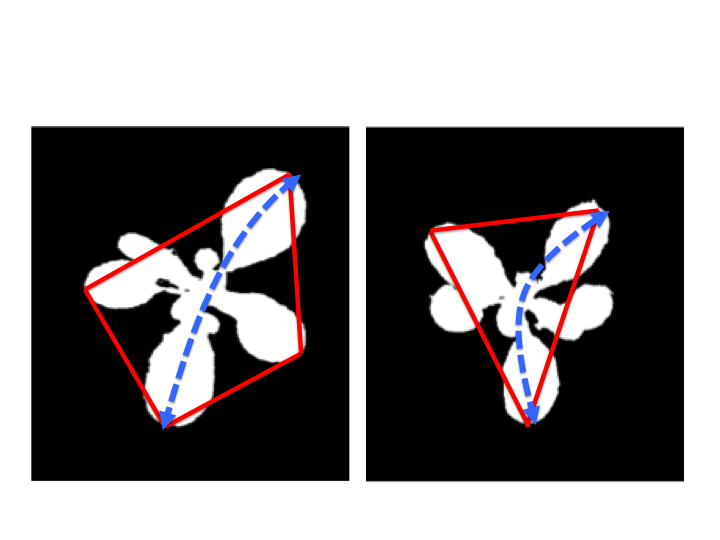


**C**

Fig. S7. Analysis of phenotypes (PA, CA, PL) with principal component analysis (PCA) of non-irradiated and irradiated (200 Gy) populations and selected represented phenotypes from the populations. Principal component analysis (PCA) of phenotypes (PA, CA, PL) of *Arabidopsis*. Panel A: PCA result from the main study. Selected image of wild type plant colored as green square box and 200 Gy plant as purple square box. Panel B: PCA result from the validation study. Panel C: Comparison of geometry shapes (red lines) of WT (left) and 200 Gy (right). Blue dash lines indicate relative angles between the first two true leaves.

Table S1. Comparison of multiple digital phenotyping measurements in three trials (preliminary, main, and validation) of gamma-rays at 15, 19, 20 days after sowing (DAS). Values in the same column followed by a different letter are significantly different (P < 0.05). PA: Projected area.

| DAS | Experiment | Treatment (Gy) | PA (cm^2^) |
| --- | --- | --- | --- |
| 15 | Main | 0 (Control) | 1.8462 a |
|  | Validation | 0 (Control) | 1.7761 a |
|  | Main | 200 | 1.3929 b |
|  | Validation | 200 | 1.2707 b |
|  | Preliminary | 200 | 1.0648 c |
| 19 | Main | 0 (Control) | 7.0382 a |
|  | Validation | 0 (Control) | 6.4722 a |
|  | Main | 200 | 5.4545 b |
|  | Validation | 200 | 5.3860 b |
|  | Preliminary | 200 | 3.8693 c |
| 20 | Main | 0 (Control) | 9.4500 a |
|  | Validation | 0 (Control) | 8.1769 b |
|  | Main | 200 | 7.2894 c |
|  | Validation | 200 | 7.1878 c |
|  | Preliminary | 200 | 5.0795 d |

Table S2. Comparing absolute growth rate (AGR) of four irradiated *Arabidopsis* populations on multiple days after sowing (DAS).

| DAS | Treatment (Gy) | Absolute growth rate |
| --- | --- | --- |
| 14 | Control | 0.4091 |
|  | 100 | 0.3834 |
|  | 200 | 0.2737 |
|  | 300 | 0.1474 |
|  | 400 | 0.0357 |
| 20 | Control | 2.0632 |
|  | 100 | 2.1189 |
|  | 200 | 1.8277 |
|  | 300 | 1.0890 |
|  | 400 | 0.2452 |
| 23 | Control | 2.7743 |
|  | 100 | 3.0151 |
|  | 200 | 2.6083 |
|  | 300 | 2.0705 |
|  | 400 | 0.4774 |
