# Supplementary material for: High-Throughput Phenotyping (HTP) Data Reveal Dosage Effect at Growth Stages in Arabidopsis thaliana Irradiated by Gamma Rays
Source: Plants (Basel). 2020 Apr 27;9(5):557. doi: 10.3390/plants9050557 (PMC7284948; doi:10.3390/plants9050557)
Supplement: Supplementary file 1 [file plants-09-00557-s001.zip › Supplementary Table S3_proof.docx]

Table. S3. Summary of phenotyping data from the preliminary, main, and validation study.

| **Experiment** | **Irradiation source** | **Irradiation doses** | **Number of tray** | **Number of samples per tray** | **DAS** | **Average of projected area (cm^2^)** | **Average of convex hull area (cm^2^)** | **Average of perimeter length (cm)** |
| --- | --- | --- | --- | --- | --- | --- | --- | --- |
| Preliminary | First batch | 200 Gy | 2 | 50 | 14 | 0.885093083 | 4.636284259 | 9.114198813 |
| Preliminary | First batch | 200 Gy | 2 | 50 | 15 | 1.084472752 | 4.859537593 | 9.205592499 |
| Preliminary | First batch | 200 Gy | 2 | 50 | 16 | 1.526874888 | 5.509312079 | 9.674961024 |
| Preliminary | First batch | 200 Gy | 2 | 50 | 17 | 2.108734478 | 6.145139654 | 10.04983548 |
| Preliminary | First batch | 200 Gy | 2 | 50 | 18 | 2.889173595 | 6.824638268 | 10.48150678 |
| Preliminary | First batch | 200 Gy | 2 | 50 | 19 | 3.964115106 | 8.033548933 | 11.18209062 |
| Preliminary | First batch | 200 Gy | 2 | 50 | 20 | 5.167017462 | 9.604751439 | 12.15333849 |
| Preliminary | First batch | 200 Gy | 2 | 50 | 22 | 8.721678508 | 15.23084203 | 14.87071177 |
| Preliminary | First batch | 200 Gy | 2 | 50 | 23 | 10.66702211 | 18.03028558 | 16.04129009 |
| Preliminary | First batch | 200 Gy | 2 | 50 | 24 | 12.49067019 | 20.64602332 | 17.06987899 |
| Preliminary | First batch | 200 Gy | 2 | 50 | 25 | 14.51557304 | 22.76105776 | 18.07076352 |
| Preliminary | First batch | 400 Gy | 2 | 50 | 14 | 0.246229949 | 1.842772179 | 6.078465005 |
| Preliminary | First batch | 400 Gy | 2 | 50 | 15 | 0.317097061 | 1.979166556 | 6.169466057 |
| Preliminary | First batch | 400 Gy | 2 | 50 | 16 | 0.453042688 | 2.258527157 | 6.523653251 |
| Preliminary | First batch | 400 Gy | 2 | 50 | 17 | 0.633633877 | 2.829261509 | 7.222772711 |
| Preliminary | First batch | 400 Gy | 2 | 50 | 18 | 0.908111721 | 3.241716471 | 7.693432024 |
| Preliminary | First batch | 400 Gy | 2 | 50 | 19 | 1.29579653 | 3.74590343 | 8.017133681 |
| Preliminary | First batch | 400 Gy | 2 | 50 | 20 | 1.78223314 | 4.367803393 | 8.607787393 |
| Preliminary | First batch | 400 Gy | 2 | 50 | 22 | 3.486129 | 6.52632968 | 10.04340042 |
| Preliminary | First batch | 400 Gy | 2 | 50 | 23 | 4.587338605 | 8.288065391 | 11.08973167 |
| Preliminary | First batch | 400 Gy | 2 | 50 | 24 | 5.830638915 | 10.08780336 | 12.09095111 |
| Preliminary | First batch | 400 Gy | 2 | 50 | 25 | 7.284049613 | 12.3082424 | 13.20532891 |
| Preliminary | First batch | 600 Gy | 1 | 50 | 14 | 0.048511002 | 1.120808147 | 4.982372278 |
| Preliminary | First batch | 600 Gy | 1 | 50 | 15 | 0.06152933 | 1.11868181 | 5.076604258 |
| Preliminary | First batch | 600 Gy | 1 | 50 | 16 | 0.078081048 | 1.287254683 | 5.267618376 |
| Preliminary | First batch | 600 Gy | 1 | 50 | 17 | 0.10249748 | 1.552131249 | 5.673176479 |
| Preliminary | First batch | 600 Gy | 1 | 50 | 18 | 0.134793314 | 1.62680906 | 5.81284499 |
| Preliminary | First batch | 600 Gy | 1 | 50 | 19 | 0.172924218 | 1.627020588 | 6.064686701 |
| Preliminary | First batch | 600 Gy | 1 | 50 | 20 | 0.223020989 | 1.737710708 | 5.890823143 |
| Preliminary | First batch | 600 Gy | 1 | 50 | 22 | 0.296120501 | 1.952963236 | 6.20976394 |
| Preliminary | First batch | 600 Gy | 1 | 50 | 23 | 0.349296052 | 1.963835484 | 6.239043034 |
| Preliminary | First batch | 600 Gy | 1 | 50 | 24 | 0.390420179 | 1.958683532 | 6.056948329 |
| Preliminary | First batch | 600 Gy | 1 | 50 | 25 | 0.417643764 | 2.069812483 | 6.408548954 |
| Main | Second batch | 0 Gy | 4 | 32 | 14 | 1.35938062 | 7.751316981 | 12.55548433 |
| Main | Second batch | 0 Gy | 4 | 32 | 15 | 1.924409531 | 8.610421107 | 12.842044 |
| Main | Second batch | 0 Gy | 4 | 32 | 16 | 2.768960815 | 9.783506367 | 13.39248988 |
| Main | Second batch | 0 Gy | 4 | 32 | 17 | 3.993738188 | 11.18766936 | 13.96672301 |
| Main | Second batch | 0 Gy | 4 | 32 | 18 | 5.444137991 | 13.21269884 | 14.65116464 |
| Main | Second batch | 0 Gy | 4 | 32 | 19 | 7.333046529 | 16.23884677 | 15.98599335 |
| Main | Second batch | 0 Gy | 4 | 32 | 20 | 9.474069857 | 19.63444112 | 17.37082868 |
| Main | Second batch | 0 Gy | 4 | 32 | 21 | 11.81877659 | 23.17730296 | 18.63355877 |
| Main | Second batch | 0 Gy | 4 | 32 | 22 | 14.16894796 | 26.82851982 | 19.813489 |
| Main | Second batch | 0 Gy | 4 | 32 | 23 | 16.70415328 | 30.29908553 | 20.89712316 |
| Main | Second batch | 0 Gy | 4 | 32 | 24 | 17.99992108 | 31.81020131 | 21.43066498 |
| Main | Second batch | 100 Gy | 3 | 32 | 14 | 1.272098076 | 7.652453739 | 12.68571803 |
| Main | Second batch | 100 Gy | 3 | 32 | 15 | 1.849705755 | 7.896828055 | 12.63480981 |
| Main | Second batch | 100 Gy | 3 | 32 | 16 | 2.676494033 | 9.121752571 | 13.24070328 |
| Main | Second batch | 100 Gy | 3 | 32 | 17 | 3.884400044 | 11.52814152 | 14.35585688 |
| Main | Second batch | 100 Gy | 3 | 32 | 18 | 5.326749576 | 13.66806024 | 15.14597302 |
| Main | Second batch | 100 Gy | 3 | 32 | 19 | 7.103786488 | 16.25488404 | 16.14986299 |
| Main | Second batch | 100 Gy | 3 | 32 | 20 | 9.26412849 | 19.62821373 | 17.27446702 |
| Main | Second batch | 100 Gy | 3 | 32 | 21 | 11.62576808 | 23.08693677 | 18.53440168 |
| Main | Second batch | 100 Gy | 3 | 32 | 22 | 14.16190056 | 26.13702887 | 19.48814825 |
| Main | Second batch | 100 Gy | 3 | 32 | 23 | 16.6305727 | 29.16944821 | 20.38233666 |
| Main | Second batch | 100 Gy | 3 | 32 | 24 | 17.81135286 | 30.73448976 | 20.92946613 |
| Main | Second batch | 200 Gy | 3 | 32 | 14 | 0.919657381 | 7.767647692 | 12.59890195 |
| Main | Second batch | 200 Gy | 3 | 32 | 15 | 1.335225845 | 8.697919052 | 13.14401918 |
| Main | Second batch | 200 Gy | 3 | 32 | 16 | 1.983906337 | 9.84725921 | 13.69611806 |
| Main | Second batch | 200 Gy | 3 | 32 | 17 | 2.826414482 | 10.65218649 | 13.8680456 |
| Main | Second batch | 200 Gy | 3 | 32 | 18 | 4.054800712 | 12.37754653 | 14.54858402 |
| Main | Second batch | 200 Gy | 3 | 32 | 19 | 5.657322689 | 14.40443786 | 15.39021505 |
| Main | Second batch | 200 Gy | 3 | 32 | 20 | 7.429692444 | 17.02067361 | 16.35380002 |
| Main | Second batch | 200 Gy | 3 | 32 | 21 | 9.428049292 | 20.04125881 | 17.49002108 |
| Main | Second batch | 200 Gy | 3 | 32 | 22 | 11.6701528 | 23.42699487 | 18.66965515 |
| Main | Second batch | 200 Gy | 3 | 32 | 23 | 14.31850435 | 27.0939754 | 19.89550605 |
| Main | Second batch | 200 Gy | 3 | 32 | 24 | 15.82643924 | 29.02797054 | 20.29057378 |
| Main | Second batch | 300 Gy | 3 | 32 | 14 | 0.472185961 | 8.160235471 | 13.02343618 |
| Main | Second batch | 300 Gy | 3 | 32 | 15 | 0.681387268 | 8.518477124 | 13.25822884 |
| Main | Second batch | 300 Gy | 3 | 32 | 16 | 0.996025474 | 8.621642813 | 13.30838964 |
| Main | Second batch | 300 Gy | 3 | 32 | 17 | 1.43084175 | 8.886961328 | 13.405178 |
| Main | Second batch | 300 Gy | 3 | 32 | 18 | 2.069935894 | 10.2507809 | 14.02270262 |
| Main | Second batch | 300 Gy | 3 | 32 | 19 | 2.899017651 | 11.17275845 | 14.31183758 |
| Main | Second batch | 300 Gy | 3 | 32 | 20 | 4.004589368 | 12.46328883 | 14.81636996 |
| Main | Second batch | 300 Gy | 3 | 32 | 21 | 5.435002246 | 14.51341881 | 15.61104038 |
| Main | Second batch | 300 Gy | 3 | 32 | 22 | 6.884707776 | 16.37246109 | 16.28479114 |
| Main | Second batch | 300 Gy | 3 | 32 | 23 | 8.808640063 | 18.84179069 | 17.05269514 |
| Main | Second batch | 300 Gy | 3 | 32 | 24 | 10.30709495 | 21.26239821 | 18.06579187 |
| Main | Second batch | 400 Gy | 2 | 32 | 14 | 0.090413143 | 4.663149089 | 10.99787799 |
| Main | Second batch | 400 Gy | 2 | 32 | 15 | 0.130910754 | 5.276179796 | 11.39515018 |
| Main | Second batch | 400 Gy | 2 | 32 | 16 | 0.209721948 | 5.509446084 | 11.42781135 |
| Main | Second batch | 400 Gy | 2 | 32 | 17 | 0.307121534 | 5.508105022 | 11.08211324 |
| Main | Second batch | 400 Gy | 2 | 32 | 18 | 0.472280729 | 5.832647684 | 11.67280136 |
| Main | Second batch | 400 Gy | 2 | 32 | 19 | 0.730053104 | 5.884356406 | 11.79587695 |
| Main | Second batch | 400 Gy | 2 | 32 | 20 | 0.916593033 | 6.395075669 | 11.87063659 |
| Main | Second batch | 400 Gy | 2 | 32 | 21 | 1.251724834 | 6.512351836 | 11.94980278 |
| Main | Second batch | 400 Gy | 2 | 32 | 22 | 1.638580262 | 7.348819701 | 12.51936679 |
| Main | Second batch | 400 Gy | 2 | 32 | 23 | 2.195787945 | 8.795479673 | 13.11241314 |
| Main | Second batch | 400 Gy | 2 | 32 | 24 | 2.605284187 | 9.20867665 | 13.45769527 |
| Validation | Second batch | 0 Gy | 4 | 32 | 14 | 1.256628445 | 6.473496084 | 10.732389 |
| Validation | Second batch | 0 Gy | 4 | 32 | 15 | 1.811042542 | 7.529981725 | 11.48249968 |
| Validation | Second batch | 0 Gy | 4 | 32 | 16 | 2.623379846 | 8.755611465 | 12.33261438 |
| Validation | Second batch | 0 Gy | 4 | 32 | 17 | 3.655072646 | 9.612463257 | 12.6801028 |
| Validation | Second batch | 0 Gy | 4 | 32 | 18 | 5.027884794 | 11.45222274 | 13.63665507 |
| Validation | Second batch | 0 Gy | 4 | 32 | 19 | 6.675174765 | 14.2777291 | 14.91165263 |
| Validation | Second batch | 0 Gy | 4 | 32 | 20 | 8.402693926 | 16.87637894 | 16.05077728 |
| Validation | Second batch | 0 Gy | 4 | 32 | 21 | 10.30248731 | 19.32167917 | 16.9100229 |
| Validation | Second batch | 0 Gy | 4 | 32 | 22 | 12.26814855 | 22.09765357 | 17.90710146 |
| Validation | Second batch | 0 Gy | 4 | 32 | 23 | 14.16066509 | 24.94827241 | 18.90376435 |
| Validation | Second batch | 200 Gy | 4 | 32 | 14 | 0.995036738 | 7.258529006 | 11.76697585 |
| Validation | Second batch | 200 Gy | 4 | 32 | 15 | 1.455142179 | 8.064139507 | 12.04932443 |
| Validation | Second batch | 200 Gy | 4 | 32 | 16 | 2.136918317 | 9.14865702 | 12.61648564 |
| Validation | Second batch | 200 Gy | 4 | 32 | 17 | 2.994985903 | 9.638310716 | 12.83021484 |
| Validation | Second batch | 200 Gy | 4 | 32 | 18 | 4.165923526 | 11.23768909 | 13.60941956 |
| Validation | Second batch | 200 Gy | 4 | 32 | 19 | 5.61507812 | 13.18895581 | 14.44303361 |
| Validation | Second batch | 200 Gy | 4 | 32 | 20 | 7.34712954 | 15.71729035 | 15.55240835 |
| Validation | Second batch | 200 Gy | 4 | 32 | 21 | 9.210286066 | 17.913321 | 16.39946165 |
| Validation | Second batch | 200 Gy | 4 | 32 | 22 | 11.05506051 | 20.31211095 | 17.2842975 |
| Validation | Second batch | 200 Gy | 4 | 32 | 23 | 12.70617474 | 22.6060958 | 18.13636183 |
| Validation | Second batch | 300 Gy | 4 | 32 | 14 | 0.455919901 | 6.123416259 | 10.62695028 |
| Validation | Second batch | 300 Gy | 4 | 32 | 15 | 0.65793453 | 6.430351435 | 11.00051339 |
| Validation | Second batch | 300 Gy | 4 | 32 | 16 | 0.936187521 | 6.369646405 | 11.00652679 |
| Validation | Second batch | 300 Gy | 4 | 32 | 17 | 1.304884595 | 6.824322867 | 11.33221165 |
| Validation | Second batch | 300 Gy | 4 | 32 | 18 | 1.835753494 | 7.607535306 | 11.72623305 |
| Validation | Second batch | 300 Gy | 4 | 32 | 19 | 2.49094301 | 7.719470076 | 11.43327776 |
| Validation | Second batch | 300 Gy | 4 | 32 | 20 | 3.371816213 | 8.723498873 | 12.12262835 |
| Validation | Second batch | 300 Gy | 4 | 32 | 21 | 4.404623099 | 10.34713468 | 12.91303868 |
| Validation | Second batch | 300 Gy | 4 | 32 | 22 | 5.598988281 | 11.94460722 | 13.66230665 |
| Validation | Second batch | 300 Gy | 4 | 32 | 23 | 6.721076609 | 13.38538289 | 14.25187381 |
